# Renal replacement therapy in the intensive care unit

**DOI:** 10.4103/0972-5229.45078

**Published:** 2008

**Authors:** Jose Chacko

**Affiliations:** **From:** Consultant, Multidisciplinary Intensive Care Unit, Manipal Hospital, Bangalore, India

**Keywords:** Acute renal failure, renal replacement therapy

## Abstract

Acute renal failure is a frequent complication in critically ill patients that carries with it considerable morbidity and mortality. The management of renal failure in patients with multi-organ failure is different from that of renal failure that presents as a single organ failure. Intermittent haemodialysis, done in the conventional manner may not be tolerated by most critically ill patients. Continuous renal replacement therapy is physiologically superior; however, there is lack of strong evidence to prove a clinical benefit. Hybrid therapies that combine the benefits of intermittent haemodialysis and continuous therapies have emerged in the past few years. These are simpler to carry out, provide more flexibility and may be cost effective and need to be studied in a systematic manner.

## Introduction

Acute renal failure is a frequent complication in critically ill patients and carries a mortality of 50 to 70%.[1] The traditionally held belief has been that kidney failure does not kill on its own as long as complications such as hyperkalaemia, acidosis and volume overload are prevented. There is evidence to suggest that this may not always be the case. We know today that acute renal failure in the critical care setting may be an independent predictor of mortality.[2,3] The management of acute renal failure in the setting of multi-organ failure is considerably different from that of renal failure as a single organ failure (e.g.; due to nephrotoxic drugs). The traditional criteria for initiating dialysis only in the face of diuretic resistant volume overload, metabolic acidosis or hyperkalaemia are largely unsuited to the management of acute renal failure in modern intensive care practice. There is strong evidence to suggest that early and more intense renal replacement therapy (RRT) can result in improved survival in the critically ill patient.[4,5] Although a recent trial did not show superiority of intensive RRT compared with more conventional treatments, continuous renal replacement therapy (CRRT) and 3 days per week of conventional haemodialysis were combined in one arm, making the results difficult to interpret.[6] This review focuses on current concepts in defining acute renal failure, its optimal management in the intensive care setting and the available evidence in regard to commonly employed modalities of RRT.

## The “RIFLE” criteria

The Acute Dialysis Quality Initiative (ADQI) has put forward a new classification for stratification of acute renal dysfunction.[7] This classification is meant to provide a uniform definition for Acute Kidney Injury similar to the consensus criteria for SIRS and ALI / ARDS. The severity of acute kidney injury increases from class “R” to “E” [Table 1]. Worsening kidney injury by the RIFLE criteria has been shown to increase mortality.[8]

**Table 1:** The RIFLE criteria for classification of Acute Kidney Injury

Class	GFR criteria	Urine output criteria
**R**isk	Creatinine × 1.5 or GFR decrease > 25%	Urine output < 0.5 mls/kg × 6 hours
**I**njury	Creatinine × 2 or GFR decrease > 50%	Urine output < 0.5 mls/kg × 12 hours
**F**ailure	Creatinine × 3 or GFR decrease > 75%, creatinine > 4.0 mg/dl or acute rise of > 0.5 mg/dl	Urine output < 0.3 mls/kg × 24 hours or anuria × 12 hours
**L**oss	Complete loss of renal function for > 4 weeks	
**E**nd Stage	> 12 weeks	
Renal Disease		

## When to initiate RRT in the ICU

Conventional criteria for initiation of RRT include volume overload, metabolic acidosis, hyperkalaemia, uraemic encephalopathy, pericarditis, etc. These are clearly appropriate triggers to initiate therapy in stable patients with renal failure presenting as a single organ failure. However, in sick patients with multiorgan failure, RRT should probably be begun much earlier; however, the exact timing of initiation of therapy depends on individual clinical circumstances. In haemodynamically unstable, oliguric patients, it will obviously be difficult or impossible to administer fluid resuscitation to meet their requirements if RRT and appropriate volumes of fluid removal cannot be employed. Provision of nutritional support in a highly catabolic state would also be facilitated if early RRT is initiated. However, survival benefit with the use of early RRT has not been clearly demonstrated. Bouman *et al*. randomised 106 patients with AKI into 3 arms-early high volume, early low volume and late low volume haemofiltration.[9] The early group received treatment for urine output < 30 mls/hr for 6 hours and creatinine clearance < 20 mls/mt; while the late group received treatment if plasma urea level was more than 40 mg/dl, K more than 6.5 and severe pulmonary oedema. There was no difference in 28 day survival or renal recovery between the groups. However, the small sample size was not powered to demonstrate a survival benefit.

## Dialysis modalities

### Intermittent haemodialysis (IHD)

This has been the conventional mode of renal replacement therapy. By this method, solute clearance takes place by diffusion across a semi-permeable membrane. Blood is allowed to pass through a bundle of hollow fibres, surrounded by dialysate fluid. The dialysate fluid typically has a pH and electrolyte compostion similar to plasma and runs opposite to the direction of blood flow on the outside of the fibres. Molecules with a higher concentration in the blood diffuse through the membrane in to the dialysate fluid. The rate of transfer depends on the concentration gradient, the molecular size and the permeability of the membrane. Membranes are designed to limit the size of the molecules that can be transferred across. This is to prevent the movement of higher molecular weight solutes such as proteins and peptides.

IHD is initiated with a blood flow rate of 150 to 200 mls/mt and increased gradually, up to 500 mls/mt. The dialysate flow rate is usually set around 200 to 300 mls/mt to start with, up to a maximum of 500 mls/mt. Increasing blood and dialysate flow rates will increase clearance, but the increase is not proportional to the rise in flow rates. In critically ill patients, hypotension is common at the initiation of IHD. Haemodynamic instability may be attenuated by starting with low blood and dialysate flows and titrating upwards as tolerated. IHD is typically done for about four hours, 3 to 4 times a week.

IHD, practiced in the manner described is very efficient and safe in the patient with end stage renal disease. However, several problems may arise when this modality is applied to the critically ill patient in multi-organ failure. Hypotension is a commonly encountered problem with IHD; this can be immediately life threatening and may be an added insult to the recovering kidneys.[10] In addition, the control of volume overload and uraemia can only be episodic with IHD. Thus, conventional IHD can often be a hazardous undertaking in the critically ill patient with multi-organ failure. However, once haemodynamic stability is attained and if the patient continues to be dialysis dependent, IHD may be the preferred modality. Profound and rapid osmotic shifts within the brain can happen during IHD leading to brain swelling;[11,12] hence it is relatively contraindicated in conditions like hepatic encephalopathy where cerebral oedema and raised intracranial pressure is a concern.

### Continuous Renal Replacement Therapies

**Figure 1:**
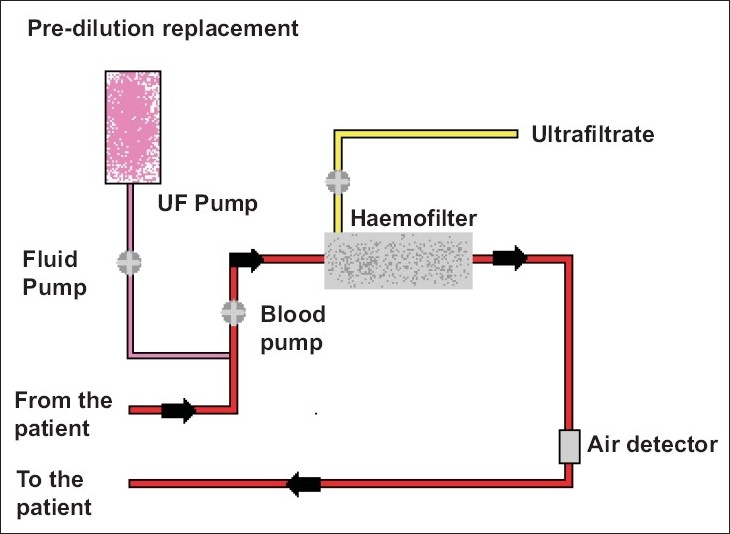
Continuous Veno-venous haemofiltration. Patient's blood is passed through a haemofilter. The ultrafiltration and replacement rate are controlled by roller pumps

The haemodynamic instability that is often associated with IHD along with the risk of injury to the recovering kidney led to the evolution of continuous renal replacement therapies (CRRT). Kramer *et al* first described the technique of continuous arterio-venous haemofiltration, using the patient's blood pressure to drive blood through the haemofilter. The rate of ultrafiltration was controlled by adjusting the height of the drainage bag.[13] Blood flow rate depended on the patient's blood pressure; this was often low in hypotensive patients. Continuous veno-venous techniques using double lumen catheters inserted into a central vein have supplanted these early arterio-venous techniques [Figure 1].

CRRT results in continuous control over solutes, acid-base and electrolyte balance and removes fluid in a slow, controlled fashion according to patient requirements. There are several methods of doing CRRT, based on the mechanism of clearance.

### Continous Veno-venous Haemofiltration (CVVH)

With this technique solute clearance is through convection or “solvent drag”. As fluid filters through the membrane, it “drags” solutes along with it. The volume of ultrafiltration depends on the transmembrane pressure, permeability, membrane thickness, surface area and pore size. Small and middle sized molecules are cleared by convection. The volume of fluid ultrafiltered is usually about 1-3 L/ hr; this is substituted with a “clean” replacement fluid with an appropriate electrolyte concentration. The replacement fluid can be administered pre or post filter. Pre-filter administration helps in prolonging the life of the filter by reducing the viscosity of the fluid that enters the filter; however this might also result in marginally reduced clearance. Post-filter administration concentrates blood inside the filter, resulting in an increased gradient and theoretically, might improve clearance at the expense of possible reduced filter life. Uchino *et al* in their study of 48 patient involving 309 filters showed predilution was a significant independent predictor of increased filter life. Pre-dilution also resulted in a reduction in the heparin dose and higher platelet counts. No favourable changes on the daily creatinine or urea levels were observed with the post-dilution technique.[14]

### Continuous Veno-venous Haemodialyis (CVVHD)

With this modality, a dialysate fluid is run countercurrent to the blood flow. The dialysate flow is set below the blood flow rate, usually 1-3 L/hr, unlike IHD, where the dialysate flow is higher. Ultrafiltrate is set according to requirement. Clearance occurs mainly by diffusion; however, if the ultrafiltrate flow is set high, some amount of convective clearance may also occur. Diffusion being the primary mechanism, only the small molecular weight solutes are cleared. Clearance of intermediate sized molecules like the cytokines is poor.

Diffusive and convective clearance can be combined by using high ultrafiltrate volumes with replacement fluid and adding countercurrent dialysate flow as well, at an appropriate rate (CVVHDF).

### Practical Management of CRRT

In practice, a double lumen catheter is inserted into the internal jugular or femoral vein. The subclavian vein is usually avoided because of the high incidence of stenosis or thrombosis that would render the ipsilateral arm unusable for the creation of an AV fistula in case long term dialysis is required.[15] For long term use, tunneled catheters with a dacron or silver impregnated collagen cuff is preferable. Internal jugular catheters with the proximal end curved downward allows easier fixation and is more comfortable for the patient. High flux, biocompatible membranes such as polyacrylonitrile and polysulfone membranes are preferred. CRRT is usually initiated with a blood flow rate of 100mls/mt and gradually increased up to 200mls/mt. In CVVH, the ultrafiltrate volume is usually set around 1 to 3 litres/hr. Ronco *et al* showed in a randomised controlled trial that ultrafiltrate volumes of 35mls/kg/hr are superior to 20 or 45mls/kg/hr.[5] In an average adult, this would be around 2.5L/hr. Replacement fluid is adjusted based on the rate of fluid removal required - this would depend on the haemodynamic and volume status of the patient. High volume haemofiltration using high flux membranes is an area of ongoing interest. Although Inflammatory mediators such as interleukin-1β, interleukin-6, interleukin-8 and other middle molecules that mediate sepsis may be effectively removed by haemofiltration,[16] whether such therapy leads to improved outcomes is not clear. An ongoing randomised controlled trial involving 35 ICUs in Australia and New Zealand is currently recruiting patients to compare outcomes between normal (25mls/kg) versus “augmented” (40mls/kg) ultrafiltrate volumes.

### Slow Continuous Ultrafiltration (SCUF)

Fluid removal at a constant rate is targeted with this therapy. No dialysate or replacement fluid is used; hence solute clearance is negligible. Fluid removal is set between 100 to 300 mls /hr depending on the haemodynamic status of the patient. This is very effective therapy when fluid removal is the only goal in patients who are not azotaemic. Patients who would benefit from SCUF are those with fluid overload that is resistant to diuretic therapy as in refractory cardiac failure or in patients with ARDS who require fluid removal. Excess fluid removal may benefit by improving gas exchange as well as haemodynamic parameters.

### Anticoagulation during CRRT

RRT involves passing the patient's blood though plastic tubings and membranes. This results in triggering of the clotting as well as the complement cascade. IHD can be done without any systemic anticoagulation, but CRRT usually requires some degree of anticoagulation to prevent frequent clotting of filters and down time that would significantly reduce of the efficacy of treatment. In practice, the circuit is rinsed with saline, containing 5000 to 20,000 units of heparin. A bolus dose of 500 to 1000 units is given, followed by an infusion of 5-10 units/kg/hr. An APTT of 30 to 45 seconds may be optimal.[17] APTT measurements must be done every 6 hourly for monitoring efficacy of anticoagulation with heparin. It may not be safe to use heparin in post-operative patients and those who are at a high risk of bleeding due to other reasons. There are several options available to prolong filter life in such situations. Prostaglandin I2 or E1 may be used in place of heparin. The prostaglandins work by inhibition of platelet aggregation, sparing the normal coagulation mechanism. They also cause vasodilatation, that might cause systemic hypotension. Fiaccadori *et al*,[18] studied 51 patients undergoing CVVH using prostacyclin 4ng/kg/mt as a continuous infusion pre-filter. The mean circuit life was 15 hrs with 4 instances of major bleeding. Hypotension requiring fluids or pressors occurred in 15.5% of CVVH sessions. The authors concluded that prostacycline carries low risk of haemorrhagic complications while allowing maintenance of filter patency to carry out effective CRRT. If systemic anticoagulation is not advisable (e.g.; post operative patients), regional anticoagulation can be considered by giving heparin pre-filter and reversing it with protamine post-filter.[19] It is also possible to provide regional anticoagulation with sodium citrate pre-filter calcium post-filter.[20] The citrate chelates calcium ions which are co-factors at multiple steps of the coagulation cascade. Metabolic alkalosis can occur during citrate anticoagulation as the citrate gets converted to bicarbonate. Hypocalcaemia may develop if calcium supplementation is inadequate as citrate causes chelation of calcium ions. Hypomagnesaemia can also occur due to chelation;[21] however this is uncommon probably because magnesium shifts from the intracellular to the extracellular compartment. The high magnesium content of bicarbonate buffered solutions may also help prevent hypomagnesaemia. Hence it is crucial to monitor ionised calcium, magnesium and acid-base status at regular intervals during citrate calcium anticoagulation. Other modes of anticoagulation using low molecular weight heparin, danaparoid and hirudin have also been described. If no form of anticoagulation is possible, normal saline at the rate of 50 to 100 mls/hr can be used to flush the circuit to maintain filter patency. This can add to the fluid intake and needs to be taken into account while calculating fluid removal. It is also important to use as large-bored a catheter as possible, as frequent filter clotting may often be associated with sluggish flows. Administration of replacement fluids pre-filter also might prolong filter life.

### Filter life

The filter and and the venous chamber are the two most common sites of clot formation. Anticoagulation as described previously, will play an important role in maintaining circuit patency. The maintenance of adequate blood flow is another crucial factor that prevents premature clotting. Increased negative pressure on the arterial side and positive pressure on the venous side could mean an early sign of clotting and would necessitate action. Change of limb or neck position could impede flow and needs constant vigilance and repositioning if required. Rinsing the circuit with normal saline containing 5,000 to 20,000 units of heparin may be effective in prolonging circuit life.[22] Polysulfone membranes may be less thrombogenic compared to polyacrylonitrile membranes.[23] Administration replacement fluid as predilution might also enchance circuit patency without any adverse effect on clearance.[14] Venous chamber clotting may be prevented by keeping the blood level almost full, moving it down at the earliest sign of clot formation, adding post-dilution fluid to this chamber and priming it with heparin.[24]

### Cost effectiveness of CRRT

AKI requiring CRRT in the critical care setting adds considerably to the cost of care. The cost of CRRT involves the filter and circuit as well as the cost of large volumes of fluid that is required for this mode of therapy. If the circuit life is short, the costs multiply several times. Personnel involved with providing CRRT need special training as well as ongoing educational programs to keep their skills updated. However, cost effectiveness depends to a large extent on clinical outcomes. Although the cost of IHD may be considerably cheaper compared to CRRT in the short term, the short and long term clinical outcomes would need to be taken in to account. Although a definite survival benefit has not been shown with CRRT compared with IHD in the critical care setting, there is some evidence that CRRT might result in a lower incidence of end stage kidney disease. Thus, it is possible that the possible long term advantage of a higher rate of renal recovery might make CRRT more attractive in economic terms as well.[25]

### Slow Low Efficiency Daily Dialysis (SLEDD)

Continuous therapies tend to be more complex, associated with the requirement for anticoagulation and costlier, with the requirement for high volumes of fluid. The lack of flexibility to move patients for procedures, interventions etc while on continuous therapies is also a disadvantage. This has resulted in the increasing use of “hybrid” therapies that try to match the physiological advantages that CRRT offers. SLEDD involves the use of blood and dialysate flows significantly less than that used with conventional IHD. Typically, blood flow rates of around 100 to 200 mls/mt and dialysate flows of less than 300 mls/mt is used. Treatment time is extended to 6 to 12 hours every day. This results in slower solute clearance and fluid removal and results in haemodynamic stability that may be comparable to CRRT. SLEDD therapies are also less expensive as there is no requirement for large volumes of customised fluids. Besides, there is greater flexibility in terms of the ability to move patients out of the intensive care unit for investigations or interventions by allowing a scheduled down time.

## Continuous Vs Intermittent therapies - what's the evidence?

The physiological superiority of CRRT over conventional IHD is unquestioned. Does this result in improved clinical outcomes? Many trials that compare these modalities involve patients with different severity of illness at baseline; crossover from one arm to the other was also allowed in many studies, making it difficult to interpret the results. Besides, the majority of trials do not include patients with significant haemodynamic instability - precisely the subgroup of patients who are likely to have a survival advantage with continuous therapies. Kellum *et al* did a meta-analysis of 13 clinical trials involving 1400 patients.[26] Out of this, only 3 were randomised. On unadjusted analysis, there was no difference survival between CRRT and IHD. However, when adjusted for study quality or baseline severity of illness, or both, CRRT was associated with improved survival. Under no conditions, either of inclusion criteria or adjustment method did CRRT fare worse. Mehta *et al*, in their study, randomised 166 patients to receive either IHD or CRRT.[27] CRRT was associated with increased ICU and hospital mortality. However, patients in the CRRT group had a higher baseline severity of illness by APACHE II and III scores, more hepatic failure and more organ failures. There was strong bias in favour of IHD by all these counts, thus obviating meaningful analysis. In a more recent trial, Vinsonneau *et al*, for the haemodiafe group compared alternate day IHD with CRRT and reported no survival benefit at 60 days. However, the trial did not standardise the time of initiation of therapy or the dose of delivered dialysis. The actual CRRT dose delivered was only 25 ml/kg/hr, significantly less than the optimal 35 ml/kg/hr that has been associated with improved outcomes.[28]

SLEDD holds the promise of combining the advantages of continuous therapy with the inherent simplicity of haemodialysis. Initial trials with SLEDD have been promising.[29] Slow low efficiency diafiltration (SLEDD-*f*) by combining SLEDD with ultrafiltration has been shown to provide stable renal replacement therapy with low molecular weight solute removal that is comparable with CRRT or IHD and possibility of large molecular weight clearance.[30]

## Who should be responsible for RRT in the ICU - intensivist or nephrologist?

In India, most ICUs follow an “open” structure and the skills required for RRT may not be readily available within the unit. Nephrologists are involved early in the management of patients with AKI and contribute to their care. RRT in the ICU is mostly carried out by technicians from the nephrology department. However, critical care nephrology as a subspecialty is fast emerging and critical care physicians are likely to be more intricately involved with the management of patients who develop AKI. It is also likely that critical care nursing would emerge as a nursing subspecialty with RRT in critical care as part of the curriculum.

## Summary

Acute renal failure is an independent predictor of mortality in the ICU. IHD, as applied in the conventional manner is largely unsuited to the sick ICU patient with multiorgan failure. CRRT is clearly superior to IHD in regard to physiological end points - clinical outcomes have not been adequately studied in patients with equal baseline severity of illness. CRRT is inherently complex with the requirement for anticoagulation and the use of high volumes of fluid and is much costlier compared to IHD. Modifications of IHD, with low blood and dialysate flows, extending it to 6 to 12 hours and administering it on a daily basis has resulted in several forms of “hybrid” therapy. Such therapies are able to combine the advantages of both IHD and CRRT. In effect, modifications of conventional IHD has made it similar to CRRT in many ways. There is a pressing need to study “hybrid” therapies for further evaluation related to clinical outcomes.
